# MicroRNA-Gene Expression Network in Murine Liver during *Schistosoma japonicum* Infection

**DOI:** 10.1371/journal.pone.0067037

**Published:** 2013-06-25

**Authors:** Pengfei Cai, Xianyu Piao, Shuai Liu, Nan Hou, Heng Wang, Qijun Chen

**Affiliations:** 1 MOH Key Laboratory of Systems Biology of Pathogens, Institute of Pathogen Biology, Chinese Academy of Medical Sciences & Peking Union Medical College, Beijing, People's Republic of China; 2 Key Laboratory of Zoonosis, Ministry of Education, Institute of Zoonosis, Jilin University, Changchun, People's Republic of China; 3 Department of Microbiology and Parasitology, Institute of Basic Medical Sciences, Chinese Academy of Medical Sciences & School of Basic Medicine, Peking Union Medical College, Beijing, People's Republic of China; Instituto de Salud Carlos III, Spain

## Abstract

**Background:**

Schistosomiasis japonica remains a significant public health problem in China and Southeast Asian countries. The most typical and serious outcome of the chronic oriental schistosomiasis is the progressive granuloma and fibrosis in the host liver, which has been a major medical challenge. However, the molecular mechanism underling the hepatic pathogenesis is still not clear.

**Methodology and Principal Findings:**

Using microarrays, we quantified the temporal gene expression profiles in the liver of *Schistosoma japonicum*-infected BALB/c mice at 15, 30, and 45 day post infection (dpi) with that from uninfected mice as controls. Gene expression alternation associated with liver damage was observed in the initial phase of infection (dpi 15), which became more magnificent with the onset of egg-laying. Up-regulated genes were dominantly associated with inflammatory infiltration, whereas down-regulated genes primarily led to the hepatic functional disorders. Simultaneously, microRNA profiles from the same samples were decoded by Solexa sequencing. More than 130 miRNAs were differentially expressed in murine liver during *S. japonicum* infection. MiRNAs significantly dysregulated in the mid-phase of infection (dpi 30), such as mmu-miR-146b and mmu-miR-155, may relate to the regulation of hepatic inflammatory responses, whereas miRNAs exhibiting a peak expression in the late phase of infection (dpi 45), such as mmu-miR-223, mmu-miR-146a/b, mmu-miR-155, mmu-miR-34c, mmu-miR-199, and mmu-miR-134, may represent a molecular signature of the development of schistosomal hepatopathy. Further, a dynamic miRNA-gene co-expression network in the progression of infection was constructed.

**Conclusions and Significance:**

This study presents a global view of dynamic expression of both mRNA and miRNA transcripts in murine liver during *S. japonicum* infection, and highlights that miRNAs may play a variety of regulatory roles in balancing the immune responses during the development of hepatic pathology. The data provide robust information for further researches on the pathogenesis and molecular events of hepatopathy induced by schistosome eggs.

## Introduction

Schistosomiasis, a debilitating disease, caused by agents of the genus *Schistosoma* afflicts more than 230 million people worldwide (http://www.who.int/mediacentre/factsheets/fs115/en/index.html). The pathology of chronic infection with *S. japonicum* or *S. mansoni* has been well known as hepatosplenic schistosomiasis, with clinical symptoms of granuloma formation, periportal fibrosis, portal hypertension, hepatosplenomegaly, ascites, and the formation of vascular shunts [1], [2]. The granulomatous responses likely induced by soluble schistosomal egg antigens (SEA) are regarded as self-protective reaction of the infected hosts. In murine schistosomiasis, schistosomal eggs lodged in the liver release SEA, which stimulates CD4^+^ T cells-mediated immune infiltration, granuloma formation, and eventual fibrosis [2], [3]. Earlier studies found that more severe granulomatous responses were induced by the infection with *S. japonicum* than *S. mansoni*, indicating that the histopathology and perhaps the etiology of the lesions, were different between the two species [4]. Recently, the temporal gene transcriptional profiles in liver and spleen tissues after the onset of egg deposition in the *S. japonicum*-infected murine model have been investigated [5], [6]. However, the gene expression profiles and gene regulatory mechanisms, particularly the association between mRNAs and miRNAs, at different stages of infection have not yet been fully explored.

MiRNAs are one of the most important classes of endogenous non-coding small RNAs of ∼22-nucleotide in length found in most eukaryotes, which could modulate activity of gene expression at post-transcriptional level [7], [8]. Currently, a large number of miRNAs have been reported in mammalian cells [9]. Among them, a set of miRNAs have been confirmed to play fundamental roles in gene regulation in various liver diseases, such as HBV and HCV-related hepatitis [10], liver fibrosis [11], NAFLA [12], [13], ALD [14], and HCC [15]–[18]. So far, the miRNA expression profiles within host liver and their functionalities associated with the development of schistosomal hepatopathy, which is a peculiar type of chronic liver disease differing from hepatic cirrhosis, remains unclear, though the miRNA profiles of schistosome itself during the parasite development have been extensively characterized [19]–[21]. Investigations on these aspects will help us better understand the molecular mechanisms of schistosomal hepatopathy. Recently, Han *et al.* have presented the miRNA expression profiles in different tissues of BALB/c mice in the early phase (10 dpi) of *S. japonicum* infection, which may be pathologically irrelevant [22].

Major advances have been achieved in high-throughput sequencing technology, which enables us to detect the expression profiles of small RNA in various tissues or organisms with a relatively low cost. The aim of the current study was to investigate the gene expression profiles in the liver after *S. japonicum* infection and the possible gene regulation mechanisms. By combination of RNA-Seq with microarray hybridization, we could simultaneously identify the differentially expressed mRNA and miRNA transcripts in the liver of *S. japonicum*-infected mice, which will be helpful for predicting potential targets of miRNAs and defining the miRNA-mRNA regulatory network; all of these would be in favor of revealing the relationship between gene expression alternation and the regulatory miRNAs, and the molecular mechanisms involved in the development of schistosomal hepatopathy. Meanwhile, the differentially expressed miRNAs in the initial phase of infection could serve as novel diagnostic markers for schistosomiasis, while miRNAs involved in granulomatous inflammation and fibrosis in the mid-late stages of infection may have potential to be novel intervention targets.

## Results and Discussion

### Differentially Expressed Genes in Murine Liver during*S. japonicum* Infection were Associated with the Progression of Hepatopathy

We systematically investigated the mRNA transcriptome in murine liver during the progression of *S. japonicum* infection with a whole genome microarray platform. In total, after normalization, 1594, 2580, and 5194 genes were found differentially expressed (Fold change > = 2 or < = 0.5) in the liver tissues of infected mice at 15, 30, and 45 day post infection (dpi), respectively, compared with the uninfected control. Of which 743, 1434, and 2361 genes were up-regulated, while 851, 1146, and 2833 genes were down-regulated at the corresponding time points (Table S1, S2, S3). With the progression of the parasite development in the host and the development of hepatic pathology, the number of differentially expressed genes significantly increased. This was more obvious in the late phase of infection (45 dpi), when hepatic egg burden and the manifestation of granulomatous responses and associated fibrosis have dramatically increased [5]. The results were in-line with the previous transcriptomic studies which found that more than 5,000 genes in mouse liver showed differential expression patterns 4 weeks post *S. japonicum* infection [5], [6]. Though significant transcriptomic alternations were accompanied with the onset of egg-laying in the liver, profiling changes were observed before the maturation of the parasite, as early as 15 dpi.

### Genes and Gene Clusters with Differential Expression Patterns during Infection

**Figure 1:**
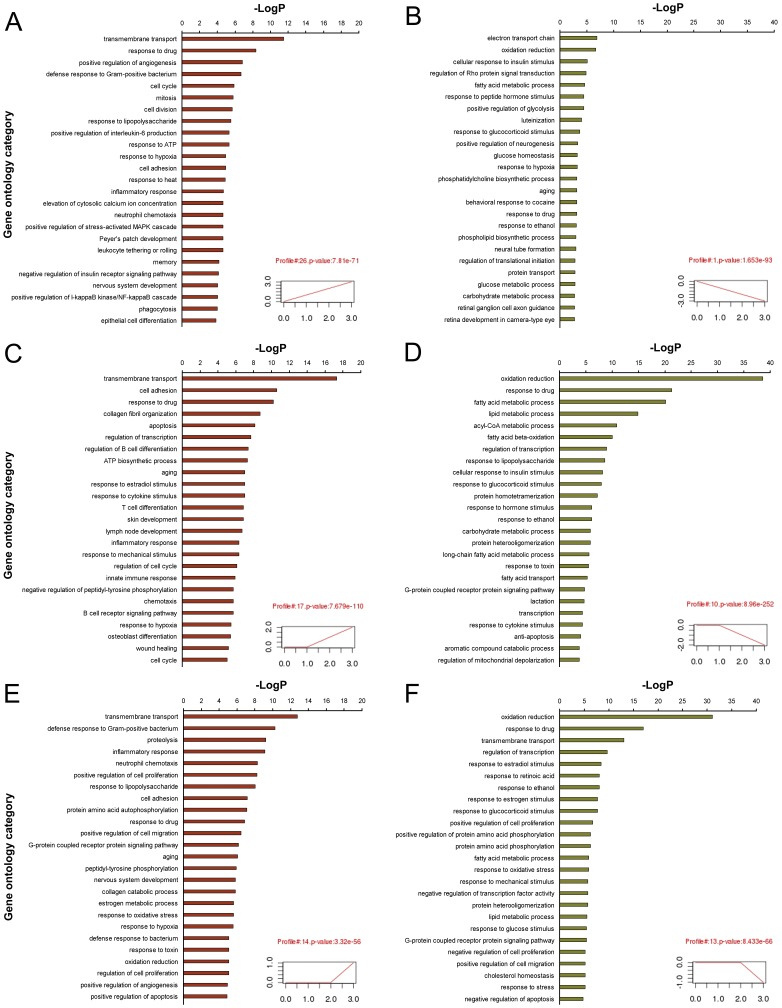
GO category analysis of significant cluster profiles in murine liver during*S. japonicum* infection. (*P* value <0.05, FDR <0.05, only the top 25 GO categories were shown). (A and B) The significant GO categories for consistently up-regulated (cluster profile 26) and down-regulated (cluster profile 1) genes during infection, respectively. (C and D) The significant GO categories for up-regulated (cluster profile 17) and down-regulated (cluster profile 10) genes followed by egg deposition, respectively. (E and F) The significant GO categories for up-regulated (cluster profile 14) and down-regulated (cluster profile 13) genes in the late phase of infection, respectively.

In order to profile the differentially expressed genes at the three time points after parasite infection and screen for the significant gene clusters with similar expression patterns associated with the progression of hepatopathy, we first defined 26 profiles with temporal expression patterns based on the trend of expression changes during the course of infection. STEM software was employed to determine the temporal expression pattern and significance of the differentially expressed genes. In total, ten significant clusters (profiles 1, 2, 4, 10, 13, 14, 17, 23, 25, and 26) were identified *(p*<0.05/N, N = 26) (Figure S1 and Table S4). As the infection progresses, the genes in profiles 26 and 1 responded rapidly in the early stage (15 dpi). Genes in profile 26 were gradually up-regulated, while genes in profile 1 were consistently down-regulated. Profile 17 and 10 represented the gene association that responded in the mid-phase of infection (30 dpi). Genes in profiles 17 and 10 were up-regulated and down-regulated, respectively, with the onset of egg deposition in the organ. Genes in profile 14 were up-regulated; while genes in profile 13 were down-regulated at the late infection stage after severe hepatic granuloma formation (45 dpi).The genes assigned in significant clusters were subjected to Gene Ontology (GO) analysis for functional classification (Table S5). The top 25 GO categories of cluster profiles 26, 1, 17, 10, 14, and 13 were shown in Figure 1.

In profile 17, several categories, for example, regulation of B and T cell differentiation, response to cytokine stimulation, inflammatory response, innate immune response, chemotaxis, B cell receptor signaling pathway, and collagen fibril organization, were more prominent (Figure 1C), indicating that extensive immune responses, such as the crucial switch from a moderate Th1 to a vigorous Th2-dominant response and the recruitment of regulatory T cells to the inflammatory site, have been mounted against the activating stimuli of egg-derived antigens at this time point. During the late infection (45 dpi), genes within the categories of neutrophil chemotaxis and positive regulation of cell migration were overexpressed (profile 14, Figure 1E), which was coincident with the migration of granulocytes and macrophages to the periphery of granuloma. Meanwhile, genes within the categories of inflammatory response and collagen catabolic process were also significantly up-regulated at this time point, which was likely related to the increasing granulomatous inflammation and fibrosis in the liver of the infected mice [5]. On the other hand, suppression of hepatic oxidation (oxidation reaction) and metabolic function (fatty acid and sugar metabolic process) already emerged even in the early infection, both of which were more severely repressed in the late infection stage (Figure 1B, D, and F).

### Network of Host Gene Responses during the Development of Schistosomal Hepatopathy

**Figure 2:**
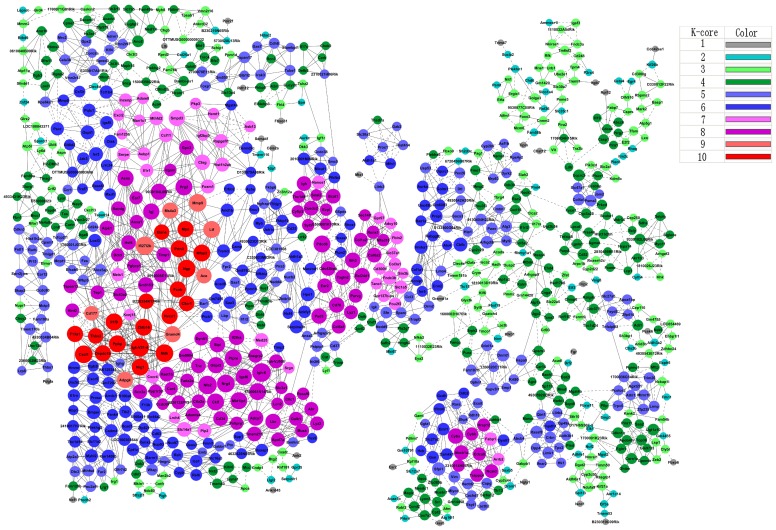
Dynamic-Gene expression network. Genes from significant differentially expressed profiles based on SCT analysis were analyzed and identified by gene co-expression network with k-core algorithm. Cycle nodes represent genes, the size of nodes represents the power of the interrelation among the nodes, and real edges between two nodes represent positive regulations between genes, while virtual edges between two nodes represent negative regulations between genes. Color of cycle represents the different k-core value. The higher the k-core value, the more central status the genes occurs within the network. In the current study, the top k-core value is 10, which is regarded to have a core status within the global gene network.

In order to discern the major gene functions associated with the development of schistosomal hepatopathy, Dynamic gene co-expression network during infection were constructed (Figure 2 and Table S6). In total, more than 900 genes were identified to involve into the network. Among them, 19 genes were identified to play crucial regulatory roles in the Dynamic-Gene Co-expression network (Degree > = 10 and k-core > = 10) (Table 1). Igk-V21-4, the immunoglobulin kappa chain variable 21 (V21)-4, was the top one with highest degree of interaction with other genes in the Dynamic-Gene expression network. However, the expression pattern of this gene was similar to that of B-cell chemokine *CXCL13*, which peaked after the influx of CD19^+^ B-cells into the liver [5]. This result may mirror that the maturation and/or retention of activated B-cells in the liver is still likely to contribute to the progression of hepatic immunopathology, in addition to that B cell responses are required for the development of granulomatous inflammation in the early stages of *S. japonicum* infection [23].

**Table 1:** Top 19 genes identified to represent the important regulatory function in the Dynamic-Gene expression network.

Gene symbol	Gene Description	degree	k-core
Igk-V21-4	immunoglobulin kappa chain variable 21 (V21)-4	17	10
Cxcl1	chemokine (C-X-C motif) ligand 1	16	10
Mdk	Midkine	16	10
Ngp	neutrophilic granule protein	16	10
Nrg1	neuregulin-1	15	10
Ppbp	pro-platelet basic protein	15	10
Zbtb16	zinc finger and BTB domain containing 16	15	10
Mfap2	microfibrillar-associated protein 2	15	10
Prtn3	MGC:175489 proteinase 3	15	10
Il1f9	interleukin 1 family, member 9	15	10
F13a1	Coagulation factor XIII, alpha subunit	14	10
Mpo	Myeloperoxidase	14	10
Depdc1b	DEP domain containing 1B	14	10
Fcnb	ficolin B	14	10
Hvcn1	clone:Y0G0138E16	14	10
Thbs3	thrombospondin 3	13	10
Elane	elastase 2, neutrophil, mRNA	13	10
C5ar1	complement component 5a receptor 1	13	10
B230354K17Rik	adult male corpora quadrigemina cDNA	13	10

In consistent with the previous observation that a set of hepatic pathology associated genes were dynamically expressed in the procession of hepatic inflammatory infiltrate and fibrosis during *S.japonicum* infection [5], [24], we found that some of these genes, such as chemokines (e.g. Cxcl1, Cxcl2, Cxcl13, Cxcl16, Ccl3, and Ccl11), chemokine receptors (e.g. Cxcr4 and Cxcr5), and matrix metalloproteinases (e.g. MMP2, MMP8, and MMP9) were co-expressed in the network to regulate the related genes at different degrees during *S. japonicum* infection in this study (Figure 2 and Table S6). Among them, chemokine CXCL1 exhibited a peak expression at 45 dpi (∼13-fold higher than that of uninfected mice), which may be in the central location of the regulatory network during the development of schistosomal pathology (degree = 16, k-core = 10, Table 1). It has been known that CXCL1 is the chemoattractant for neutrophils and hepatic stellate cells (HSCs), and is able to induce wound healing responses of HSCs or myofibroblasts [5]. Given the importance of these cells in regulating the fibrotic responses in other liver diseases [25], our result further indicated that CXCL1 may serve as a key mediator involved in the molecular events associated to the complex fibrotic pathology induced by schistosome in the late phase of infection.

### Differentially Expressed miRNAs in Murine Liver during Infection

**Figure 3:**
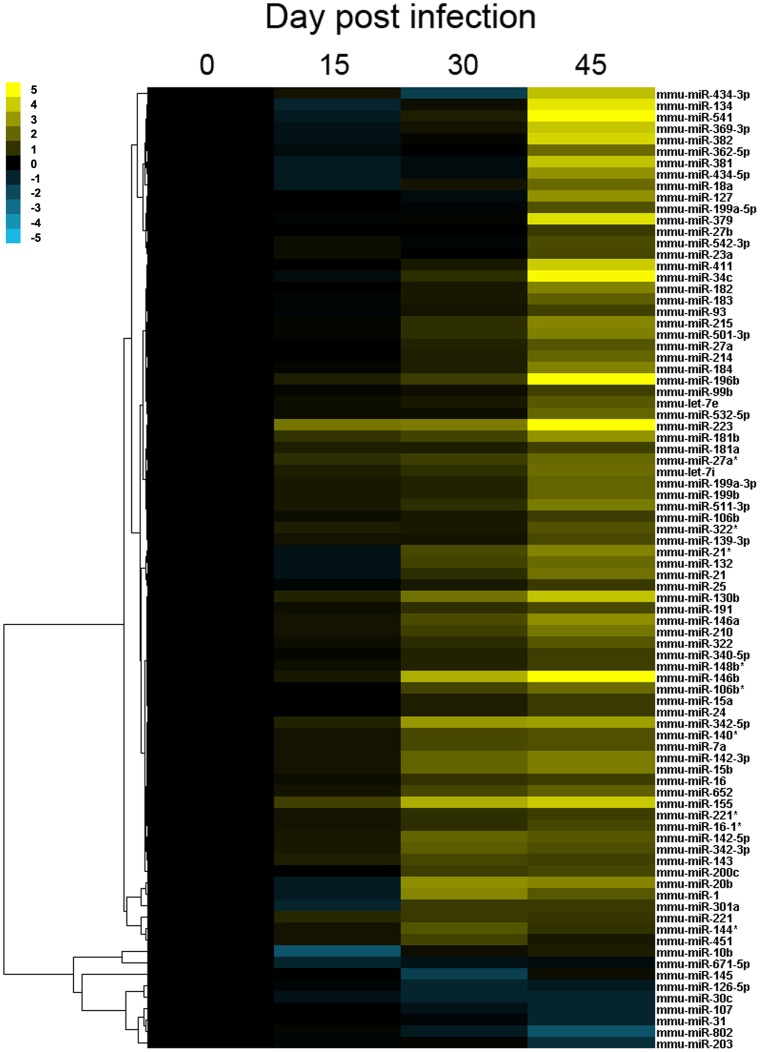
Hierarchical clustering of differentially expressed miRNAs in murine liver during the development of schistosomal hepatopathy by Cluster 3.0. Heatmap was constructed based on the log_2_ transform data of fold change of TPM value of miRNAs. The expression of most miRNAs was consistently up-regulated during infection, with significantly altered in the late phase.

Expressional alterations of hepatic miRNAs have been shown to regulate cellular processes such as liver carcinogenesis and fibrosis [26], [27]. Here, the host miRNA responses in murine liver during the progression of *S. japonicum* infection were systematically investigated. In total, about 10 million high quality reads were obtained from each library with the next generation high-throughput sequencing (Table S7). Of which, 8,055,938 (78.20%), 8,040,899 (79.79%), 6,973,994 (79.66%), and 9,004,923 (81.01%) reads in the libraries H0, H15, H30, and H45, respectively, were perfectly matched to the mouse genome sequences with a redundancy about 95% (Table S8). After blasting the perfectly matched tags to the Sanger miRBase, 580 tags homologous to the mouse mature miRNAs cataloged in the database were identified, which accounts for more than one half of all known mouse mature miRNAs. The expression level (normalized as “transcripts per million”, TPM) of each miRNA detected in the four libraries was shown in Table S9. In total, more than 130 miRNAs were differentially expressed between the experimental and control groups (Table 2). A heatmap was constructed to show the dynamic expression of these miRNAs (Figure 3).

**Table 2:** Differentially expressed hepatic miRNAs between experimental and control groups during experimental murine schistosomiasis.

MiRNA*	Max Expressed (TPM)				Fold change (infected mice/control)		
	H0	H15	H30	H45	H15/H0	H30/H0	H45/H0
mmu-miR-223	0.5	2.5	2.7	31.4	5.0	5.4	62.8
mmu-miR-196b	0.4	0.6	0.9	16.9	1.5	2.3	42.3
mmu-miR-146b	33.3	47.5	360.5	1206.6	1.4	10.8	36.2
mmu-miR-541	5.1	3.1	7.5	168	0.6	1.5	32.9
mmu-miR-34c	21.4	17.7	40.4	657.2	0.8	1.9	30.7
mmu-miR-134	8.3	4.2	9.9	189.6	0.5	1.2	22.8
mmu-miR-379	49	43.3	52.8	1044.4	0.9	1.1	21.3
mmu-miR-382	3.1	2.2	3.4	57.4	0.7	1.1	18.5
mmu-miR-411	2.7	2.7	3.9	43.8	1.0	1.4	16.2
mmu-miR-155	1.6	3.9	16.9	25.1	2.4	10.6	15.7
mmu-miR-369-3p	0.7	0.5	0.9	10.5	0.7	1.3	15.0
mmu-miR-381	2.5	1.6	1.9	35.6	0.6	0.8	14.2
mmu-miR-130b	1	1.6	4.7	14.2	1.6	4.7	14.2
mmu-miR-434-3p	1.2	1.6	0.4	16.3	1.3	0.3	13.6
mmu-miR-342-5p	1.6	2.5	12.8	14.1	1.6	8.0	8.8
mmu-miR-181b	3.6	7.3	8.9	26.9	2.0	2.5	7.5
mmu-miR-434-5p	2.4	1.4	1.9	17.7	0.6	0.8	7.4
mmu-miR-127	8.7	8.8	6.9	63.9	1.0	0.8	7.3
mmu-miR-146a	409	514.6	1165	2814.9	1.3	2.8	6.9
mmu-miR-20b	1.6	1	11.2	9.9	0.6	7.0	6.2
mmu-miR-215	13	14.1	24.9	80.1	1.1	1.9	6.2
mmu-miR-21*	2	1.4	5.4	12	0.7	2.7	6.0
mmu-miR-182	43.2	41.4	61.4	254.6	1.0	1.4	5.9
mmu-miR-184	13.2	14.7	19.9	77.5	1.1	1.5	5.9
mmu-miR-501-3p	14.3	15.5	27.4	81.8	1.1	1.9	5.7
mmu-miR-142-3p	76.7	101.7	296.5	424.8	1.3	3.9	5.5
mmu-miR-15b	7	9	27.4	38.4	1.3	3.9	5.5
mmu-miR-511-3p	2.5	3.5	4.7	13.2	1.4	1.9	5.3
mmu-miR-210	5.1	6.5	12.3	25.9	1.3	2.4	5.1
mmu-miR-21	13893.7	9415	26609	65900.1	0.7	1.9	4.7
mmu-let-7i	157	230.7	304.1	697.6	1.5	1.9	4.4
mmu-miR-362-5p	16.5	13.9	17.1	71.7	0.8	1.0	4.3
mmu-miR-106b*	3.1	3.1	7.9	13.4	1.0	2.5	4.3
mmu-miR-132	5	3.4	12.3	21.1	0.7	2.5	4.2
mmu-miR-18a	7.3	4.7	9.8	30.6	0.6	1.3	4.2
mmu-miR-27a*	7.3	13.7	16.8	30.1	1.9	2.3	4.1
mmu-miR-214	5.2	5.2	7.6	20.8	1.0	1.5	4.0
mmu-miR-532-5p	97.3	113.2	121.5	386.9	1.2	1.2	4.0
mmu-miR-199a-3p	64.7	91.3	101.2	256.7	1.4	1.6	4.0
mmu-miR-199b	64.7	91.3	101.2	256.7	1.4	1.6	4.0
mmu-miR-652	18.6	25	47.5	69.4	1.3	2.6	3.7
mmu-miR-183	25.7	24.3	34.7	95.6	0.9	1.4	3.7
mmu-miR-142-5p	20.9	28.7	80.7	69.3	1.4	3.9	3.3
mmu-let-7e	204.6	239.4	292.7	672.1	1.2	1.4	3.3
mmu-miR-322	15.6	18.4	28.4	51	1.2	1.8	3.3
mmu-miR-1	16.1	10.4	99.2	52.6	0.6	6.2	3.3
mmu-miR-27a	104.6	100.7	162.6	333.4	1.0	1.6	3.2
mmu-miR-322*	6.1	9	8.7	18.9	1.5	1.4	3.1
mmu-miR-199a-5p	12.8	12.9	12	38.9	1.0	0.9	3.0
mmu-miR-140*	903.4	1159	2408.5	2697.9	1.3	2.7	3.0
mmu-miR-7a	97.2	127.6	266.3	288.1	1.3	2.7	3.0
mmu-miR-342-3p	5.2	7.3	18.2	15.1	1.4	3.5	2.9
mmu-miR-542-3p	8.4	9.8	7.6	23.7	1.2	0.9	2.8
mmu-miR-191	1302.5	1569.4	2430.7	3528.8	1.2	1.9	2.7
mmu-miR-23a	7.9	9.1	7.6	21.4	1.2	1.0	2.7
mmu-miR-139-3p	10.2	13.2	13.2	27.1	1.3	1.3	2.7
mmu-miR-200c	49.9	51	113	132.1	1.0	2.3	2.6
mmu-miR-16-1*	4.8	6.3	9.2	12.5	1.3	1.9	2.6
mmu-miR-148b*	12.3	14.3	19.6	30	1.2	1.6	2.4
mmu-miR-143	9246.1	14007.5	24207.1	22504.5	1.5	2.6	2.4
mmu-miR-181a	51.1	78.1	77	122.6	1.5	1.5	2.4
mmu-miR-93	743.7	673.4	990.4	1774	0.9	1.3	2.4
mmu-miR-99b	693.3	781.1	810	1635.9	1.1	1.2	2.4
mmu-miR-106b	68.8	85.4	97.9	159.1	1.2	1.4	2.3
mmu-miR-221*	36.5	45.9	67.8	84.2	1.3	1.9	2.3
mmu-miR-340-5p	90.5	99.4	143.2	208	1.1	1.6	2.3
mmu-miR-16	163	194	332.7	370.1	1.2	2.0	2.3
mmu-miR-25	43.4	38.9	59.1	96.3	0.9	1.4	2.2
mmu-miR-15a	79.3	81.8	117	175.3	1.0	1.5	2.2
mmu-miR-24	301.1	290.5	460.7	665	1.0	1.5	2.2
mmu-miR-301a	1	0.5	2.2	2.2	0.5	2.2	2.2
mmu-miR-27b	813.1	833	804	1763.5	1.0	1.0	2.2
mmu-miR-221	12.2	21	26.8	25.5	1.7	2.2	2.1
mmu-miR-144*	114.8	151.4	370.5	233	1.3	3.2	2.0
mmu-miR-10b	72.1	17.3	87.5	107.7	0.2	1.2	1.5
mmu-miR-451	101	128.2	256.5	140.6	1.3	2.5	1.4
mmu-miR-145	11.5	11.4	3.9	14.1	1.0	0.3	1.2
mmu-miR-671-5p	30.5	14.8	20.4	23.3	0.5	0.7	0.8
mmu-miR-126-5p	673.3	583.1	330.9	383.3	0.9	0.5	0.6
mmu-miR-107	24.2	23.6	17.4	11.8	1.0	0.7	0.5
mmu-miR-31	71.4	70.5	61.8	34.8	1.0	0.9	0.5
mmu-miR-30c	335.7	242.3	154.9	160.1	0.7	0.5	0.5
mmu-miR-203	273.7	253.8	291.4	112.8	0.9	1.1	0.4
mmu-miR-802	54	60.8	33.8	13.1	1.1	0.6	0.2

* MiRNAs with TPM value less than 10 in all libraries were not shown.

### MiRNAs Related to the Hepatic Pathology of Schistosomiasis

At the pre-patent stage of *S. japonicum* infection, the expression pattern of miRNAs was not changed dramatically. However, several miRNAs, such as mmu-miR-146b, mmu-miR-155, mmu-miR-223, mmu-miR-142-3p, mmu-miR-15b, and mmu-miR-126-5p, were observed to be up-regulated significantly in the mid-phase of infection (30 dpi) (Table 2 and Figure 3). Intriguingly, some of these miRNAs have been characterized to be associated with inflammation responses or the expression of oncogenes [28], [29]. More importantly, miR-155, miR-146, and miR-223 have been suggested to regulate the inflammatory responses after the recognition of pathogens by the Toll-like receptors (TLRs) [30], [31]. Based on the observation of temporal changes of the cellular composition in the liver of *S. japonicum*-infected mice [5], the upregulation of mmu-miR-146b and mmu-miR-155 at this time point may reflect the recruitment and activation of B and T lymphocytes to the periphery of granulomas in response to the stimuli of antigens secreted by the eggs.

The expression of mmu-miR-146b and mmu-miR-155 was continently up-regulated in the late phase of infection, when the SEA-induced hepatic granulomatous pathology associated with fibrosis became more profound [5]. Several studies have revealed that co-activation of miR-146 and miR-155 is regulated by NF-κB signaling pathway and may facilitate a negative-feedback loop that will protect the host from an excessive TLR4 response [31], [32]. The dramatic expanding of mmu-miR-146b and mmu-miR-155 in the late phase of infection indicated that a similar role may be exerted by these miRNAs to subtly control the extent of hepatic immunopathology of schistosomiasis. Further, miR-155 has been recognized as a pleiotropic modulator in a variety of immune cells. In CD4^+^ T cells, miR-155 represses the expression of transcription factor *c-Maf*, which is likely to contribute to the attenuation of Th2 cell response [33]. Thereby, the up-regulation of mmu-miR-155 may also facilitate Th1/Th2 balance in the progression of schistosomal egg-induced immunopathology. In macrophage, up-regulation of miR-155 was found to be a common consequence after exposing to a broad range of inflammatory mediators [30]. A further study revealed that miR-155 was a mediator contributing to the Th1/Th2 equilibrium, favoring a pro-Th1/classical activation of macrophages by reducing the expression of several pro-Th2/IL-13-dependent genes [34]. IL13 was suggested as an important cytokine responsible for the development of hepatic fibrosis by inducing the alternative activated macrophages (aaMφ) [35], [36]. Here, we postulated that mmu-miR-155 may act as a negative modulator to restrict the excessive expression of pro-Th2/IL-13-dependent genes in aaMφ, and finely manage the hepatic fibrosis process in the late infection phase.

The expression of the myeloid-specific miRNA, miR-223, was intriguing for its rapid expressional alteration as early as 2-week post infection, and it was also the most dramatically up-regulated miRNA at 45 dpi. A previous study has shown that granulocytic differentiation is controlled by a regulatory circuit involving miR-223 and two transcriptional factors, NFI-A and C/EBPalpha [37]. More importantly, a loss-of-function analysis of miR-223 in murine model has revealed that this miRNA can negatively regulate progenitor proliferation and granulocyte differentiation and activation, and more evidences have been provided to support a model in which miR-223 acts as a fine-tuner of granulocyte production and the inflammatory response [38]. The remarkable upregulation of mmu-miR-223 in the late phase of *S. japonicum* infection may also fit this scenario, that is, mmu-miR-223 may prevent over-activation of granulocytes and consequently limit the magnitude of the immune responses. However, the expression of miR-223 in hepatocellular carcinoma has been found frequently repressed [39], indicating that one particular miRNA may play different roles in different hepatic diseases. It would be interesting to investigate whether the human homologues of miR-223 is also expressed in the same manner during *S. japonicum* infection, which may be of potential importance as a candidate of anti-pathology therapy.

In addition, some other miRNAs previously reported to be associated with fibrosis, such as miR-34c, miR-199, and miR-214, also exhibited a peak expression in the liver of infected mice at 45 dpi (Table 2 and Figure 3). Li *et al.* observed that the rno-miR-34 family was significantly up-regulated in dimethylnitrosamine-induced hepatic fibrosis, and suggested that miR-34 family members may be involved in the process by targeting acyl-CoA synthetase long-chain family member 1 (ACSL1) [40]. Here, the expression of mmu-miR-34c was as high as 30-fold more in the late phase of infection compared to that in the early infection, suggesting its important roles in the progression of liver pathogenesis. Further, Murakami *et al.* observed that the progression of liver fibrosis was related with over-expression of the miR-199 and 200 families in a CCL4-induced murine model [41], and miR-199a-5p was also observed to mediate TGF-β-induced lung fibroblast activation by targeting Caveolin-1 [42]. Here, we found that the expression levels of miR-199 family members were up-regulated (3–4 fold) in the liver of *S. japonicum*-infected mice at 45 dpi compared with that of uninfected mice, indicating that dysregulation of miR-199 members may represent a general mechanism contributing to the fibrotic process. Recently, Iizuka *et al.* suggested that miR-214-5p may play crucial roles in the activation of stellate cells and the progression of liver fibrosis [43]. Our result here may also support this hypothesis, as mmu-miR-214 was up-regulated ∼4-fold in the liver of mice in the late infection of *S. japonicum* compared to that of normal mice. As the hepatic stellate cells trans-differentiation into myofibroblasts has been regarded as a key event in liver fibrogenesis [44], it is reasonable to suggest that this miRNA could be a potential target of anti-fibrosis therapy if further investigation can confirm that the up-regulation of mmu-miR-214 indeed occurs in the HSCs.

### Identification of Potential Targets of Consistently Regulated miRNAs

**Figure 4:**
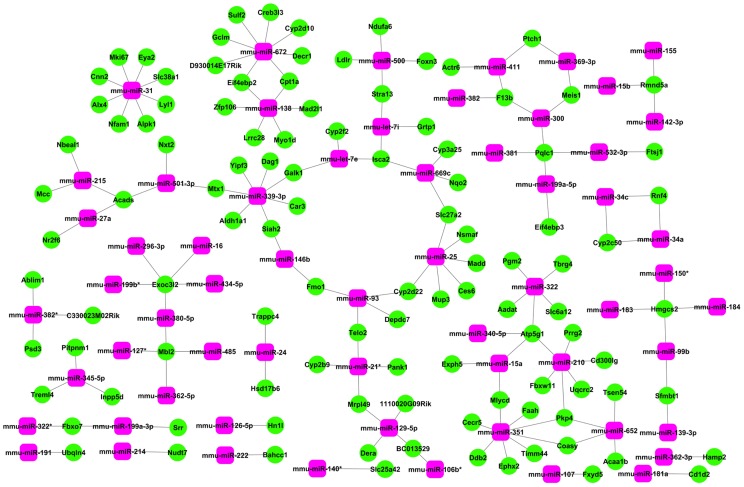
MiRNA-Gene-Network. Pink box nodes represent miRNAs, and green cycle nodes represent the predicted target genes. Edges show the inhibitory effect of miRNA to its predicted targets. Two overexpressed miRNAs (mmu-miR-351 and mmu-miR-672) show the most target mRNAs of 8 (degree 8). In contrast, mmu-miR-31 has the highest degree in those under-expressed miRNAs. Degree means the contribution one miRNA to the genes around or the contribution one gene to the miRNAs around. The key miRNAs and genes in the network always have the highest degrees.

We further defined 83 consistently up-regulated and 6 consistently down-regulated miRNAs across the progress of infection within those differentially expressed miRNAs (Table S10). We next searched for the target genes of these consistently regulated hepatic miRNAs in the miRBase and filtered for the expression interactions between miRNAs and related predicted genes (Interaction<−0.99). This resulted in definition of potential downstream targets of a total of 107 mRNAs, most of which were commonly repressed during the progression of infection and assigned as GO of hepatic metabolize and detoxification function (Table S11). The microRNA-Gene-Network was built to indicate that the key miRNAs potentially regulate the expression of hepatic genes during the progression of *S. japonicum* infection (Figure 4 and Table S12). Mmu-miR-31, mmu-miR-351, mmu-miR-672, mmu-miR-339-3p, mmu-miR-138, mmu-miR-210, mmu-miR-25, and mmu-miR-322 were found to be more prominent and may play crucial roles in the regulatory network for their degree were more than 5.

One example to illustrate the miRNA-gene regulation network is the CoA metabolic pathway. CoA is an important cofactor in about 4% of known enzymes, including more than 100 reactions involved in metabolism [45], [46]. These enzymatic reactions play important roles in the metabolism and synthesis of fatty acids, aminoacids, cholesterol, pyruvate/lactate, glucose, and Krebs cycle intermediates, and so on [47]. In this study, we observed that *Hmgcs2* (3-hydroxy-3-methylglutaryl-Coenzyme A synthase 2) which encodes an enzyme exhibiting hydroxymethylglutaryl-CoA synthase activity, is potentially regulated by mmu-miR-183, mmu-miR-184, mmu-miR-150*, and mmu-miR-99b. Another gene, *Acads*, which encodes an acyl-Coenzyme A dehydrogenase, is the putative target of mmu-miR-27a, mmu-miR-215, and mmu-let-7i. *Coasy* (Coenzyme A synthase) was predicted to be the target gene of mmu-miR-652 and mmu-miR-351. We also determined that the expression of *Mlycd* (malonyl-CoA decarboxylase) can be suppressed by mmu-miR-15a and mmu-miR-351. Further, the expression of *Accl2* (acetyl-Coenzyme A acyltransferase 1B) and *Decr1* (2,4-dienoyl CoA reductase 1) was likely inhibited by mmu-miR-652 and mmu-miR-672, respectively. Importantly, *Pank1,* which encodes a pantothenate kinase, is a key regulatory enzyme in the biosynthesis of coenzyme A. We found that this gene was regulated by mmu-miR-21* (Figure 4). Thus, it seemed that the abnormal expression of these miRNAs coordinated to regulate the genes that associated with the CoA synthesis, and further impacted the liver metabolic process during infection.

In summary, we combined the high-throughput sequencing and whole genome microarray analysis to present a global scene of dynamic expression of both mRNAs and miRNAs in murine liver during *S. japonicum* infection. A set of genes related to immune responses, especially those related to Th2 responses and fibrosis were not only prominently expressed but also coordinated the expression of other genes. A unique panel of miRNAs with dominant expression patterns associated with the development of schistosomal hepatopathy was identified for the first time, and some of which were suggested to potentially serve as candidates of anti-pathology therapy. Putative targets of miRNAs were predicted and mRNAs and miRNAs may operate coordinately in the network to contribute to the schistosomal hepatic pathology.

## Materials and Methods

### Ethical Statement

The study was reviewed and approved by the Ethical Committee of Chinese Academy of Medical Sciences. The collection of *S. japonicum*-infected *Oncomelania hupensis* is irrelevant to protection of wildlife. China has a long term schistosomiasis elimination program, and to eliminate the schistosome-infected *Oncomelania hupensis* is regarded as one of the key steps in the program. Thus, collecting snails is not restricted but encouraged.

### Parasites and Animals

The cercariae of *S. japonicum* were freshly shed under light stimulus from parasite-infected *Oncomelania hupensis* collected from Poyang Lake, Jiangxi province, China. Nine 8-weeks female BALB/c mice were randomly assigned into three groups (group I, II, and III, three mice per group) and were percutaneously infected with 30±2 cercariae of *S. japonicum*. Liver tissues were obtained from the mice of group I, II, and III at 15, 30, and 45 dpi, respectively, with normal liver tissues from three uninfected BALB/c mice as control. All procedures carried out on animals within this study were conducted following animal husbandry guidelines of the Chinese Academy of Medical Sciences and with permission from the Experimental Animal Committee of Chinese Academy of Medical Sciences with the Ethical Clearance Number IPB-2011-6.

### Total RNA Isolation

Total RNA was extracted from the mixed liver tissues (three livers at each time point) using Trizol reagent (Invitrogen, CA, USA) according to the manufacturer’s instruction. RNA quantification and quality were measured by the Nanodrop ND-1000 spectrophotometer (Nanodrop Technologies, Wilmington, DE), denaturing gel electrophoresis and Agilent 2100 Bioanalyzer (Agilent Technologies, Palo Alto, CA). Two batches of RNAs were purified and analyzed separately.

### Microarray Hybridization and Data Mining

The hybridization procedure was carried out by KangChen Bio-Tech Inc., Shanghai, China. Briefly, after DNase digestion, each total RNA sample was amplified and labeled using a NimbleGen One-Color DNA Labeling Kit (Roche-NimbleGen) and were profiled for gene expression using Roche-NimbleGen v2 array (Cat # 05543797001) in NimbleGen Hybridization System. After hybridization and washing, the processed slides were scanned with the Axon GenePix 4000B microarray scanner. Raw data were extracted as pair files by NimbleScan software (version 2.5). NimbleScan software’s implementation of RMA offers quantile normalization and background correction. The Probe level (*_norm_RMA.pair) files and Gene summary (*_RMA.calls) files were produced. The four gene summary files were imported into Agilent GeneSpring Software (version 11.0) for further analysis. Genes that have values greater than or equal to lower cut-off: 50.0 in all samples (“All Targets Value”) were chosen for data analysis. To identify the genes that are differentially expressed, we performed a Fold-Change screening between the experimental (three groups of *S. japonicum*-infected mice) and control (normal mice) groups. The threshold we used to screen up or down regulated genes is Fold Change> = 2. All the data files have been submitted to the NCBI Gene Expression Omnibus (GEO) (http://www.ncbi.nlm.nih.gov/geo/) under Accession: GSE45985.

### Series Test of Cluster (Stc) and Series Test of Cluster of Gene Ontology (Stc-GO) Analysis

Stc analysis was performed as previously described [48], [49]. Briefly, significant differentially expressed genes between the experimental and control groups were selected according to RVM (Random variance model) corrective ANOVA [50]. In accordance with different signal density change tendency of genes during infection, we identified a set of unique model expression tendencies. The raw expression values were converted into log_2_^ratio^. Using a strategy for clustering short time-series gene expression data, we defined 26 unique model profiles. The expression model profiles were related to the actual or the expected number of genes assigned to each model profile. Significant profiles have higher probability than expected by Fisher’s exact test and multiple comparison test [51], [52].

GO-Analysis was applied to the genes belong to certain specific tendencies [49], [53]. It is used to find the main function of the genes have same expression trend according to the Gene Ontology which is the key functional classification of NCBI [54]. Generally, Fisher’s exact test and 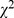 test were used to classify the GO category, and the false discovery rate (FDR) [55] was calculated to correct the *P*-value, the smaller the FDR, the small the error in judging the *P*-value. The FDR was defined as: 
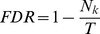
, where 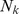 refers to the number of Fisher’s test *P*-values less than 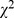 test *P*-values. We computed *P*-values for the GOs of all the differential genes. Enrichment provided a measure of the significance of the function: as the enrichment increased, the corresponding function was more specific, which helped us to find those GOs with more concrete function description in the experiment. Within the significant category, the enrichment Re was given by: 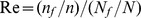, where 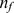 is the number of differential genes within the particular category, *n* is the total number of genes within the same category, 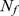 is the number of differential genes in the entire microarray, and 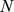 is the total number of genes in the microarray [56].

### Construction of Dynamic Gene Co-Expression Network

In order to define major gene functions during the development of schistosomal hepatopathy, a dynamic gene co-expression network was constructed as previously described [48], [57]. We selected genes from the most significant profiles, namely profiles 1, 3, 4, 10, 13, 14, 17, 20, 23, 25, and 26, as a result from Stc analysis, to construct the gene co-expression regulatory network. The normalized expression value of the Pearson correlation was transformed into measures of pairwise connection strengths [58]. The edges were then specified to feature correlation coefficients of above 0.9, to ensure strong gene co-expression relationships. Within the network, nodes represent genes, and edges between nodes depict the interaction between them. As network elements represent the regulation function of genes, global gene networks could be grouped into certain subgraphs, named k-core networks (marked with different colors), in which all genes should be connected to at least k other genes in the subgraph [59]. In light of the definition of k-core networks, core status within global gene networks consists of subgraphs that are associated with higher k-core values. All the nodes were marked with degree (represented by the size of node), which is defined as the link numbers one node has to its neighbor. Genes with higher degrees located in the centrality of the network and had a stronger capacity of regulating adjacent genes [60]. Besides, the clustering coefficient was specified to feature the density of neighbor genes of a particular gene in the network, the strength of gene co-expression relationships increased with the clustering coefficient.

### Small RNA Deep-sequencing and Configuration

Small RNA libraries were constructed and sequenced by an Illumina Genome Analyzer at the BGI (Beijing Genomics Institute, Shenzhen, China) as described previously [61]. Libraries constructed with the small RNAs extracted from liver tissue of mice at 0, 15, 30, and 45 dpi, were designated as H0, H15, H30, and H45, respectively. The raw datasets from the four libraries were pooled. Clean reads were retrieved after eliminating the adapter contamination and low quality reads. Adapter sequences were then trimmed from both ends of clean reads. All identical sequences were merged as unique tags and further mapped to the mouse genome (http://hgdownload.cse.ucsc.edu/downloads.html#mouse) with the program SOAP [62]. The perfectly matched reads were BLAST-searched against the mouse mature miRNAs deposited in Sanger miRBase (Release 16) [9], [63] using the program Patscan [64]. Expression levels of mature miRNAs from different libraries were normalized to “transcripts per million (TPM)”. Those with TPM value less than 2 in all libraries were ruled out for further analysis. The differentially expressed miRNAs were selected by a double Fold-Change screening between the test and control groups. IDEG6 [65] was used to identify miRNAs showing statistically significant difference in relative abundance between any two small RNA libraries. The general Chi-square test was applied to determine whether one particular miRNA was significantly differentially expressed between test and control samples (*P* value < = 0.01) Hierarchical clustering of the differentially expressed miRNAs was constructed by software cluster 3.0. Sequence data of the four small RNA libraries have been submitted to NIH Short Read Archive with the accession numbers of SRR796936 (for H0), SRR796957 (for H15), SRR796958 (for H30), and SRR796959 (for H45).

### MicroRNA-Gene-network

First, we defined consistently up-regulated or down-regulated miRNAs and then the mRNA targets of these miRNAs were predicted based on the Sanger database (http://microrna.sanger.ac.uk/). Next, an intersection between the targets of miRNAs and differentially expressed genes was collected. The relationship of the miRNAs and genes were counted by their differential expression values and according to the interactions of miRNAs and genes in Sanger miRBase to build the MicroRNA-Gene-Network [16], [66]. The adjacency matrix of miRNA and genes A = [ai,j] is made by the attribute relationships among genes and miRNA, and ai,j represents the relation weigh of gene i and miRNA j. In the MicroRNA-Gene-network, the circle represents gene and the square represents miRNA, and edge represents the relationship between gene and miRNA. The center of the network was represented by degree. Degree means the contribution one miRNA to the genes around or the contribution one gene to the miRNAs around. The key miRNA and gene in the network always have the biggest degrees.

## Supporting Information

Figure S1 26 unique model profiles defined in this study.
(JPG)

Table S1 Information of differentially expressed genes in the early phase of infection.
(XLS)

Table S2 Information of differentially expressed genes in the mid-phase of infection.
(XLS)

Table S3 Information of differentially expressed genes in the late phase of infection.
(XLS)

Table S4 Information of ten significant gene expression patterns.
(XLS)

Table S5 Information of all GO categories of cluster profiles 1, 2, 4, 10, 14 13, 17, 23, 25, and 26, respectively.
(XLS)

Table S6 Gene-gene relationship and properties of genes presented in Figure 2.
(XLS)

Table S7 General information of the four small RNA libraries (H0, H15, H30, and H45).
(XLS)

Table S8 Data statistics for the four small RNA libraries (H0, H15, H30, and H45).
(XLS)

Table S9 Information of all hepatic miRNAs during *S. japonicum* infection in a murine model.
(XLS)

Table S10 List of consistently up-regulated or down-regulated hepatic miRNAs during *S. japonicum* infection.
(XLS)

Table S11 GO analysis of target genes of consistently regulated hepatic miRNAs.
(XLS)

Table S12 MicroRNA-gene relationship and properties of genes and miRNAs presented in Figure 4.
(XLS)
