# Supplementary figures and images for: MicroRNA-Gene Expression Network in Murine Liver during Schistosoma japonicum Infection
Source: PLoS One. 2013 Jun 25;8(6):e67037. doi: 10.1371/journal.pone.0067037 (PMC3692539; doi:10.1371/journal.pone.0067037)

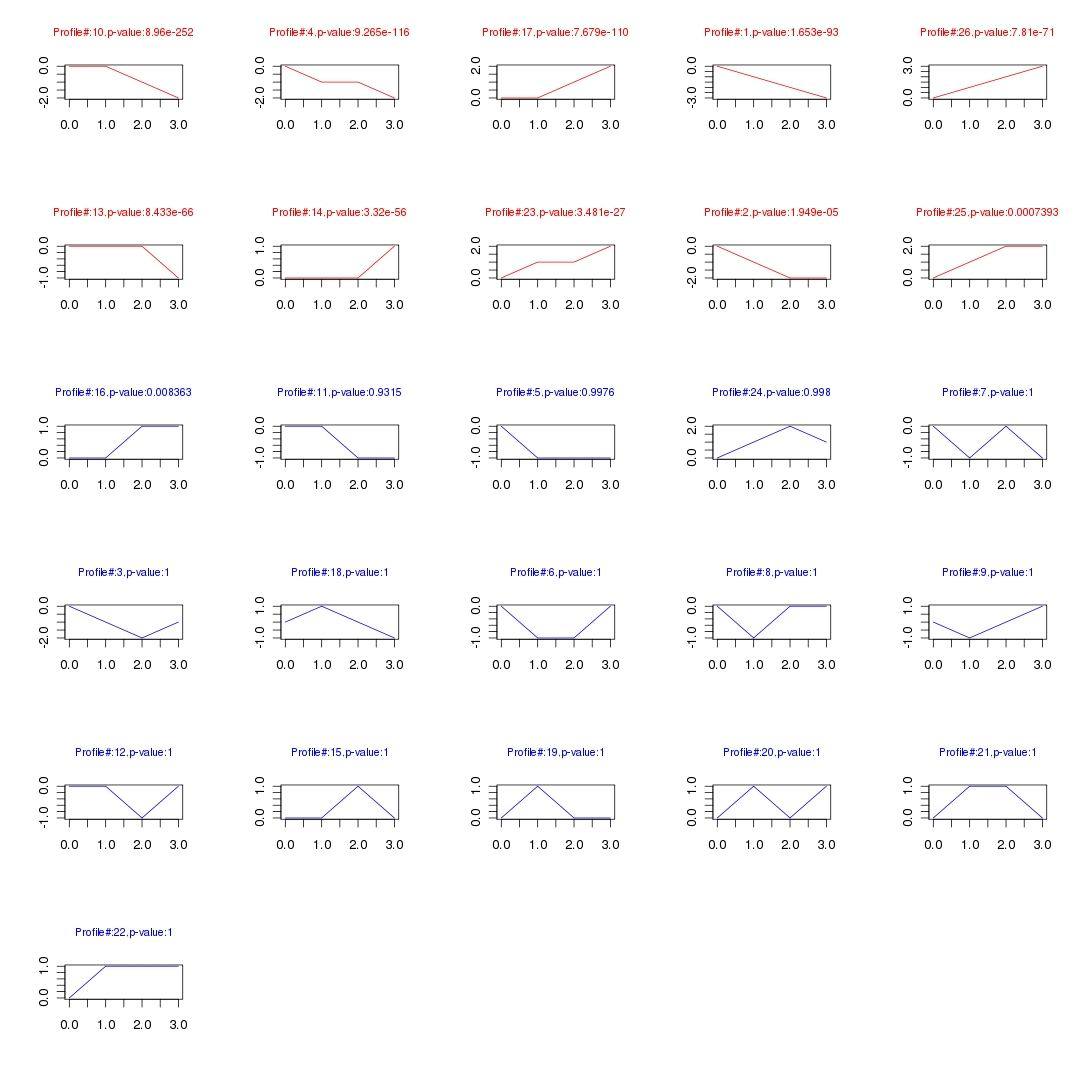

Supplement: Figure S1 — 26 unique model profiles defined in this study. (JPG) [file pone.0067037.s001.jpg]
